# Growing health in UK prison settings

**DOI:** 10.1093/heapro/day037

**Published:** 2018-05-29

**Authors:** Michelle Baybutt, Mark Dooris, Alan Farrier

**Affiliations:** Healthy and Sustainable Settings Unit, School of Community Health and Midwifery, Faculty of Health and Wellbeing, University of Central Lancashire, Brook Building 328, Preston, UK

**Keywords:** prisons, horticulture, health promotion, nature, well-being

## Abstract

Globally, prisoners tend to come from marginalized and socially disadvantaged sections of the society and exhibit a high incidence of ill health, linked to social exclusion and multiple complex needs. Prisons therefore offer an important opportunity to tackle inequality and injustice, through promoting health, reducing reoffending and facilitating community reintegration.This paper reports on and critically discusses findings from an evaluative research study, which aimed to identify and explore impacts of prisoners’ participation in an innovative social and therapeutic horticultural programme, ‘Greener on the Outside for Prisons’ (GOOP), delivered in prisons in North West England. Focus groups with 16 prisoners and semi-structured interviews with six prison staff were conducted at five sites. Presented under three overarching themes (health and well-being; skills development, employability, and work preparedness; and relationships), findings suggest that engagement with and participation in GOOP were important in improving positive mental well-being, increasing physical activity and knowledge about healthier eating; developing skills and work readiness; and building relationships and catalysing and strengthening prosocial behaviours, important for good citizenship and effective resettlement. The paper concludes that – in the context of the current UK prison reform agenda and concern about the high incidence of violence, substance misuse, self-harm and suicide – prison-based horticulture can offer multiple benefits and make a significant contribution to the creation of safe, secure, supportive and health-enhancing environments. Furthermore, it contends that by joining up health and justice agendas, programmes such as GOOP have the potential to serve as powerful catalysts for wider systemic change, thereby helping tackle inequalities and social exclusion within societies across the globe.

## BACKGROUND

### The prison context: exclusion, health and well-being

In many Western countries, the prison population has increased over the recent years, and the capacity of prison services has not kept pace (World Health Organization [WHO] Europe, 2014). Since 2000, the world prison population has grown by almost 20%, slightly above the estimated 18% increase in the world’s general population over the same period (Walmsely, 2016). This places an enormous financial burden on governments and threatens the social cohesion of societies (Penal Reform International, 2015). Overcrowding occurs in more than 20 states of the WHO European Region – including England and Wales, where the prison system has been overcrowded every year since 1994 (Prison Reform Trust, 2015). This is an obvious contributing factor to many health problems, particularly accelerating transmission of infections and having a negative impact on mental health (WHO Europe, 2014). Studies worldwide have shown that prison suicide rates are up to 10 times higher than those in the general population and that young people in prison are especially vulnerable and 18 times more likely to commit suicide than those in the wider community. A recent report (UNODC, 2014) suggests that suicide among prisoners is more frequent in Europe compared to other regions, averaging 62 per 100, 000 and accounting for 13% of all deaths in prison.

The majority of prisoners across the world are adult men (Penal Reform International, 2015). Many prisoners come from the most economically deprived and socially disadvantaged groups and exhibit multiple complex needs (Ginn, 2013). In England and Wales, the Prison Reform Trust [Prison Reform Trust (2015)] highlights strong links between offending behaviour, social exclusion and education and skills deficits – for example:

- 43% of male offenders, 32% of female offenders and 52% of young offenders were permanently excluded from school;
- 24% of men and 31% of women in prison had been in local authority care during childhood, compared to only 2% of the general population;
- 47% of prisoners report having no qualifications;
- 21% report needing help with reading, writing or numeracy and 40% with work-related skills and
- 15% of prisoner’s report being homeless prior to custody.

In addition, recent studies show that penal systems are struggling to cope with rising numbers of older prisoners. This changing prison demography is creating new pressures and particular challenges for prison healthcare services (Institute for Government, 2017). In England and Wales, male offenders aged 50 or above are the fastest growing group in prison, rising by 74 per cent in the past decade to close to 10 000, 11 per cent of the total prison population. In the UK, the over-60s population has increased eight-fold since 1990 (Penal Reform International, 2015).

Prisoners tend to lead chaotic lives, with a complex range of interconnected issues strongly linked to offending and reoffending (Baybutt and Chemlal, 2016). Research consistently demonstrates that ill-health of prisoners is higher than reported in the wider community (Senior and Shaw, 2007). Ninety per cent have mental health and/or substance abuse problems, often complicated by high-risk lifestyles, untreated chronic conditions and social issues such as homelessness, unemployment and poor levels of education. In addition to overcrowding, this is often exacerbated by violence, isolation, absence of privacy, lack of meaningful activity and inadequate health services, especially mental health (Penal Reform International, 2015). Research revealing the strong association between offending behaviour and poor health, low levels of educational attainment and wider deprivation suggests a ‘vicious cycle’ with most prisoners coming from and returning to the poorest or most socially excluded sections of society (Social Exclusion Unit, 2004). Yet 97% of offenders express a desire to stop offending and prisoners who gain employment after release are far less likely to reoffend (Prison Reform Trust, 2015).

### Promoting health in the prison setting

Prisons are not principally in the business of promoting prisoners’ health, neither does this gather political capital or public endorsement (Woodall, 2016). However, prisons have potential to make a major contribution to improving the health, well-being and life chances of some of the most marginalized and excluded individuals in the society (Baybutt *et al.*, 2006). Wider benefits of good prison health include lowering the costs of imprisonment by improving the health of the whole community, reducing public health expenditure, improving reintegration into society and reducing reoffending, reducing health inequalities and reducing the size of prison populations (WHO Europe, 2014). That said, there are important challenges. Where health promotion has been developed in prisons, it tends to follow a medical model, viewing health primarily as the absence of disease and focusing on individual lifestyle choices rather than wider determinants (Woodall *et al.* 2014b). Yet it is clear that prisoners’ health is influenced by a complexity of ‘deprivation’ and ‘importation’ factors – relating both to imprisonment itself and to circumstances pre-dating imprisonment (de Viggiani, 2006) – and that effective approaches require action on wider societal influences (Smith, 2000). Furthermore, there is a contradiction between the dominant aims and culture of prisons as a place of deterrence, punishment and reform and values central to health promotion – such as enablement and empowerment (Woodall *et al.* 2014b).

While the WHO Health in Prisons Programme has inherent challenges (Woodall, 2016), it has been widely argued that the settings approach it endorses offers opportunities to realize the potential of prisons to embrace health promotion and meaningfully tackle health inequalities (Department of Health, 2002; Baybutt and Chemlal, 2016). Appreciating that health is created and lived within settings of everyday life (WHO 1986), the approach embraces a socioecological model of health, a salutogenic orientation concerned with what creates well-being and makes people thrive, a systems perspective and a focus on holistic change (Dooris, 2013; Dooris *et al.*, 2014). Applied in this context, the settings approach prioritizes a whole prison perspective and involves revisiting notions of control, choice and empowerment and utilizing a determinants-focused framework (Baybutt *et al.*, 2006; Woodall *et al.*, 2014a, 2014b).

### Nature, gardening and health: prisons and justice settings

Reflecting insights drawn from the biophilia hypothesis, concerning humans’ innate connection with the natural world (Kellert and Wilson, 1993), a growing body of evidence highlights the positive contribution of green space and nature for physical and mental health (Maller *et al.*, 2006; Barton *et al.*, 2016; Houses of Parliament, 2016; Maxwell and Lovell, 2016; Public Health England, 2017). Contact with the natural environment improves mental wellbeing – reducing stress and improving mood, providing a restorative environment and facilitating social contact (O’Brien *et al.*, 2011; Sempik *et al.*, 2005). Studies suggest that connecting with nature can restore cognitive attention (Kaplan, 1995), positively influence blood pressure and self-esteem (Pretty *et al.*, 2007), decrease symptoms of deficit disorder (Kuo and Faber-Taylor, 2004), facilitate recovery from surgery (Ulrich, 1984) and increase perceived quality of life, strengthen community cohesion and motivate pro-environmental behaviour (Hansen-Ketchum and Halpenny, 2011). Buck [Buck (2016), p. 6] reports that evidence of the benefits of gardening for health is complex but that ‘observational and qualitative studies are consistent with a wide range of health impacts across mental and physical health and health behaviours across the life-course.’ O’Brien *et al.* [O’Brien *et al* (2011)] suggest that active hands-on engagement with nature is effective in enabling marginalized people to reintegrate into society by facilitating skills development, improving self-confidence, creating social networks, providing meaningful activity and developing a sense of responsibility. Horticulture is used around the world as means of promoting health and well-being for disadvantaged and vulnerable people in diverse contexts (Sempik *et al.*, 2005). Fieldhouse [Fieldhouse (2003)] found plant–person relationships to be immensely important to people with mental health problems, identifying the cognitive benefits of enhanced mood, reduced arousal and improved concentration and the social benefits of gardening and the focus on cooperation to achieve goals.

Lewis [Lewis (1996)] suggests that just as the interaction of human nature with green nature can enhance feelings of peace, self-esteem and restoration for people in everyday life, it can be beneficial in prison contexts. Prison-based horticultural programmes and settings offer multiple benefits – relating to skills development, behaviour and self-esteem (Flagler, 1995) and therapeutic and aesthetic respite from the wider prison, offering safe, healing places that contribute to prisoners’ survival strategies and allow staff relief from harsh workplace environments (Baybutt and Chemlal 2016; Lindemuth, 2007). Beyond the prison context, offender and nature schemes involve offenders in prison and community settings working as volunteers on nature conservation and woodland sites (Carter, 2007). Such schemes offer reparative work that benefits the public and, for offenders, provides experience of teamwork, life and skills training – while also boosting confidence and self-esteem, increasing health and well-being and aiding rehabilitation (O’Brien *et al.*, 2010).

### Greener on the outside for prisons

Informed by the above evidence, GOOP – a social and therapeutic horticulture and environmental programme – was established in 2008. It was 1 of 12 programmes funded by the National Lottery as part of Target: Well-being, a large scale public health portfolio that ran from 2007 to 2015 in North West England with three key outcome areas – mental well-being, physical activity and healthier eating.

GOOP has been run as a collaborative programme between prisons, education and health providers and a university, with prisoners choosing to participate. Drawing on the Trust for Conservation Volunteer’s (TCV) ‘Green Gym’ delivery model, which seeks to improve health through engagement with nature (Yerrell, 2008), the programme has reflected needs and opportunities offered by different types and categories of establishment, with two main forms of activity: community-based environmental ‘outworking’ and ‘in-prison’ horticultural work. The former has involved contributions to conservation and landscaping by prisoners released on temporary licence, while the latter has involved GOOP participants developing and maintaining outdoor horticultural spaces in prisons, designing new therapeutic gardens for prisoners and staff, growing food and plants and undertaking accredited training. Building on regional engagement with the Health Promoting Prisons movement (Woodall, 2016) and the WHO Health in Prisons Programme (Gatherer *et al.*, 2005), the programme’s vision was to adopt a ‘whole prison’ settings approach.

Phase 1 of GOOP ran from 2008 to 2012 in prisons across North West England, involving approximately 3500 prisoners. Subsequent funding was successfully secured, and, at the time of writing, GOOP remains operative in all public-sector prisons across the region. In order to capture learning, an evaluative research study was undertaken in the final 6 months of Phase 1, which aimed to identify and explore its impacts and benefits for participating prisoners. This paper presents and discusses key findings from this study.

## STUDY DESIGN, AIMS AND METHODS

Ethical approval was obtained from the University ethics committee, following approval for the study being granted by the Regional Custodial Services Manager. The study was conducted according to ethical guidelines with key issues including secure storage of confidential data using password-protected and/or encrypted folders and informed consent to using data from monitoring forms and quotations from interviews. Prisoners’ names were anonymized, and staff were identified only by role.

The study was informed by a socioecological model of health, which emphasizes the interconnections between environment, behaviour and well-being, recognizing the dynamic interplay between situational and personal factors (Stokols, 1996); by a psychosocial perspective, which positions the individual in networks of interpersonal relationships, organizations and social, political and economic systems (Froggett, 2002); and by insights from the Biophilia Hypothesis (Kellert and Wilson, 1993). It adopted a qualitative approach – particularly suitable for studying people within the context of organizations and for exploring their understandings and interpretations (Denzin and Lincoln, 2008). It was undertaken in five participating prisons, four adult (over 21) male and one adult female establishments (the first prisons in which GOOP was established). Researchers conducted one 40-minute focus group with prisoners at each site and additionally undertook half-hour individual semi-structured interviews with a total of five members of staff involved with GOOP, spanning all five of the study establishments. Sixteen prisoners took part in the focus groups. Prisoners were identified opportunistically through liaison with prison GOOP staff, with the main criteria for selection being that were actively involved in GOOP over a 12-week period and were willing to talk about their experiences with a researcher and other prisoners. At any one point in time, there were approximately 80 prisoners active in GOOP across the five research sites, and there were no particular difficulties in recruiting focus group participants. Staff were chosen based on their involvement with GOOP delivery or strategically with monitoring the impact of GOOP on prisoners’ health, resettlement and education. Staff who participated included gardens managers and staff and one horticultural instructor employed by the external education provider. Semi-structured interview and focus group schedules were informed by emergent findings from the overarching evaluation of Target: well-being (Phase 1) and by discussions with prison staff involved in delivering GOOP. Specifically tailored to prisoners and staff, key issues included understanding why prisoners wanted to be on the programme; examining benefits of the programme, particularly on mental wellbeing; exploring employability linked to involvement in GOOP and identifying changes that could improve the programme. Supplementary questions pursued impacts related to physical activity and healthier eating knowledge and behaviour.

All interviews and focus groups were recorded and transcribed, and prisoner and staff data were subjected to a two-stage manual thematic analysis (Braun and Clarke, 2006). It was not possible to offer participants the opportunity to check data, due to turnover of prisoners. Appreciating that the aim of the study was to explore impacts and benefits for participating prisoners, it seemed appropriate to view GOOP holistically and draw on staff perspectives on this overarching research question by integrating them alongside prisoners’ views. Prisoner-related and staff-related data were therefore subjected to a common thematic coding framework. One member of the research team attempted to discover themes within the raw data by a line-by-line analysis of verbatim transcripts and by interpreting their implications in relation to the aims of the research (O’Leary, 2004). This initial analysis and coding was cross-checked and refined by other members of the research team to produce a final report (Baybutt *et al.*, 2012).

## FINDINGS

Data are presented under three overarching themes relating to the benefits of participation in GOOP, health and well-being; skills development, employability and work preparedness and relationships. This focus on positive impacts reflects the data emerging from the interviews, although challenges and areas for improvement are noted where appropriate.

### Health and well-being

Participation in GOOP was beneficial for health and well-being in multiple ways, in part because of its holistic nature:

*It’s a gentle introduction into health and wellbeing because it’s on the physical, it’s on the mental and the social side of it. It ticks every box, does horticulture, it’s a fantastic therapy. (Horticultural Instructor)*

The hands-on nature of horticulture and the nurturing process involved impacted positively and profoundly on prisoners’ well-being and also enabled them to connect with their own journeys and experience of mental health challenges. Furthermore, tangible results attained through transforming spaces, for example from a waste ground to a functioning garden, led to an enormous sense of pride and achievement:

*When they start shooting up and growing, you start feeling that you’re getting something produced, it’s that little bit of, I don’t know – honour. (Prisoner #1)*

Linked to this, there was a growing feeling of ownership and accomplishment at having enhanced the prison environment, with knock-on benefits for other prisoners and staff:

*It’s bringing a smile to their face…They use [the garden] to have their lunch, bring their classes up to do art and do drawings…They can see the benefits and so I think they appreciate what we’re doing. (Gardens Manager)*

As GOOP gained recognition, there were reports of prison staff showcasing the gardens to visitors, and it was apparent that GOOP participants greatly valued and drew strength from the positive feedback they received, which clearly helped to build self-esteem and self-belief:

*It’s the comments that you’re getting…about how it’s improving and how it’s looking better each day…It’s just like being normal again. (Prisoner #3)*

When prisoners described how they felt when doing the work, the impact of GOOP on well-being became clearer. For those involved in activities outside their establishments, having time away from prison was in itself positively received. Furthermore, GOOP helped prisoners to reflect, put things into perspective and unwind. Contact with nature evidently played a crucial role, prompting engagement of different senses and the repeated use of words such as ‘refresh’ and ‘freshen’:

*It’s good because…we’ve all been here a long time, so it’s like, it doesn’t matter where you go you’re still in prison in your head. Coming outside, it is a totally different environment … it’s a wide open space, the smells, sounds, you know, it helps you relax and just forget. (Prisoner #4)*

As well as benefitting mental well-being, GOOP increased prisoners’ levels of physical activity through engagement in manual work:

*You’re always busy, keep[ing] fit…I’ve lost two and a half stone just working on here…It’s a workout in itself, because you’re working all the parts of your body, because you’re twisting and you’re lifting. (Prisoner #1)*

It was also clear that this exercise had knock-on effects for overall fitness and well-being, improved sleep and behaviour management and benefitted the overall prison ethos:

*They’re out all day pretty much and…they get home after work and they’re tired. So they sleep better, therefore they wake up and they’re ready to go again…it is definitely a massive bonus to their wellbeing. (Senior Manager)*

There was also evidence of GOOP encouraging healthier eating. In keeping with the whole system focus, a number of establishments used food grown as part of the programme in the prison kitchen. Prisoners and staff commented that they loved being able to eat the produce and reflected on how GOOP had resulted in a more varied and healthier diet:

*It was all organic…and every single one said how fresh and how nice [the vegetables] tasted. [And] it’s certainly raised awareness – some women have never seen some of these vegetables before…I know a lot are using [the produce] for meals and the young offenders took food back to the house to experiment with different menus. (Gardens Manager)*

More widely, it was felt that prisoners were motivated to draw on the experience. Although there was still work to be done with regard to cooking skills, prisoners showed interest in taking the experience of food growing into their future lives and sharing learning with their families:

*It tastes so much better straight from the ground, it really does. And I will certainly grow vegetables…If it’s possible I will do it because it just tastes so much better. (Prisoner #5)*

### Skills development, employability and work preparedness

At a basic level, prisoners increased knowledge of and skills for gardening and environmental conservation. They highlighted how they had begun to exchange ‘gardening tips,’ and a staff member commented on skill swapping observed between prisoners. This process was incremental, focused on the practical ‘doing’ and enhanced by being allowed to try things out and learn from mistakes:

*Two [prisoners] were already here and they had some knowledge but not a great deal…the third one who came along did have some gardening knowledge, and I think they’ve shared the knowledge from her and…they’ve learnt a lot…they’ve self-taught a great deal and I’ve let them experiment as much as they want. (Gardens Manager)*

It was also apparent that the process of engaging in horticulture and environmental activities enabled the development of wider practical and interpersonal skills:

*It’s the communication skills and interaction with other people that they never had before, it’s teamwork – that if you actually do work together, it doesn’t matter, you come to a stronger outcome. (Horticulture Instructor).*

Observations from staff were reinforced by prisoners themselves who highlighted a diversity of skills – including perseverance, concentration and mindfulness:

*You’ve got to have patience with yourself and everybody else…patience in every sense of the meaning. Patience watching things grow…and when people get on your nerves, patience with that. (Prisoner #7)*

This work experience combined with skills development and accredited training resonated with staff and prisoners alike and was understood to provide valuable preparation for post-sentence employment:

*It’s just like preparing yourself for work, what you would do on the outside. (Prisoner #3)*

Moreover, the nature of GOOP as a programme and the strong motivation and attachment it engendered resulted in a strong positive impact in terms of work ethos and preparedness to work:

*They’ve had something they’ve kind of believed in… They’ve wanted to work seven days a week and some of them wanted to work into the evening, which is a massive thing! Normally they want to get out of doing stuff, [but with GOOP] they actually want to get into doing stuff. (Senior Manager)*

Staff reflected on a wide range of specific skills developed through the various GOOP activities and how these simultaneously necessitated use of basic mathematics, reflecting that this type of learning would be rejected in more formal lessons.

While many prisoners clearly developed a passion for horticulture and environmentally focused work and said they want to go into gardening after their release, there was recognition of the wider value of skills gained and appreciation that these were transferable and could be meaningfully applied to ‘life outside’:

*I’d never touched a plant in my life, I didn’t know nothing, but I’ve learnt a hell of a lot. And I think no matter where you go…you can do it. And it sort of gives you a confidence to know that yes, I can achieve anything really. (Prisoner #7)*

Beyond employment, those interviewed highlighted the value of GOOP in developing skills enabling prisoners to continue gardening on release – both for pleasure and for utility:

*When I get out…Jobs, you know, they’re quite scarce at the moment and it’s expensive to live out there. If I’m doing this, I know that at least I could feed myself cheaply with like the vegetables and everything that I’ve learnt how to grow. (Prisoner #8)*

### Relationships

A further theme concerned relationships – between prisoners; between prisoners and staff; across the prison system and beyond prison. While leaving room for friendly rivalry between teams, GOOP encouraged prisoners to co-operate and share knowledge and skills. As intimated above, this went beyond their experience of formal prison education:

*[It’s] a team effort isn’t it? If you work individually and you work against each other, you get nothing done, because that’s harder work than working together. (Prisoner #1)*

Staff reflected that, as GOOP became established, the diversity of participants resulted in opportunities for a richer range of working relationships to be forged between prisoners with wide-ranging experiences. This focus on co-operation was seen not only to make for more harmonious living and working environments but also to prepare prisoners for release:

*I want them to interact…because one thing they will find amongst gardeners, they’ll help one another out. You go down to an allotment and you’ve never dug a hole in your life, they’ll all be round there and show you how to do it – and that’s what I try to get from them. (Gardener)*

Prisoners were overwhelmingly positive about the relationships built with staff during their participation in GOOP activities – highlighting the significance for them of feeling trusted and valued and how this allowed them to glimpse what being outside prison might feel like:

*It’s really good because obviously with having a criminal record…here it’s like, well you’re respected…and you feel like you’re in society already, coming into work is fantastic really. (Prisoner #9)*

Staff similarly reflected on the relationships with prisoners and the value of GOOP in building trust and rapport:

*If it hadn’t have been for GOOP, [the relationship] possibly would not have been quite as good and relaxed…Alright, we have to maintain order and control, but because me and none of the other staff are in uniform, the barrier’s broken down. So they’ll say things to me and the guys that they won’t probably say to the uniformed staff. (Gardens Manager)*

Most prisons involved in GOOP demonstrated a capacity to connect the horticultural work with other parts of their establishment. However, at this stage of developing the GOOP programme, only one demonstrated a ‘conscious’ whole system approach evidenced through developed relationships and joined up working – involving education, catering, residential units and external partners. It was noted how challenging it was to forge effective linkages across the regional prison system. While there were examples of prisoners transferring between establishments and continuing GOOP work, this was understood to be due to chance rather than design – and the vision of prisoners moving on and acting as mentors for new participants did not materialize. It was also apparent that participation in GOOP served as a catalyst to prisoners strengthening relationships with family members, offering a focus for developing connection and rapport:

*I write to my Nana and tell her what I’m doing because we lost my granddad a few years ago and he was the one who did all the growing…I’m hoping [my supervisor’s] going to do a photograph of my tomato plants that I can send her. She’s 87, I haven’t seen her for eight years, she’s too old to come up here. But she wrote back to me saying it’s lovely that you’re growing things. And growing the tomatoes, it reminds me of when I used to do it with my granddad. (Prisoner #10)*

Beyond the family, GOOP also offered opportunities for those prisoners engaged in projects outside prison to develop relationships with community members:

*It is a good thing because, I mean some people have been in a really long time, 25 years and…coming out is a really big thing and mostly it’s hard because you don’t really know how to act around the public…and this breaks you in gently and that’s really positive. (Prisoner #4)*

## DISCUSSION

While limited in scale and scope, exploring a sample of stakeholders’ views and perceptions at a single point in time, this study has revealed the profound and wide-ranging impacts attributed to participation in GOOP, with feedback suggesting that the data proved influential in supporting continued funding and development of the programme. Benefits encompassed all three Target: Wellbeing outcome areas, echoing literature highlighting and the essentially holistic nature of well-being (La Placa *et al.*, 2013) and of the gains accruing from gardening (Buck, 2016). With regard to physical well-being, there was evidence of increased levels of exercise, linked to enthusiasm for GOOP activities. This resulted in positive impacts for sleep patterns, acknowledged to be a challenge within prison contexts (Cope, 2003), and consequently for behaviour – with staff reporting prisoners having less pent-up energy. Participation also raised awareness of, interest in and appreciation of fresh food and, linked to this, stimulated interest in how food growing relates to the environment, thereby echoing other gardening initiatives in providing informal sustainability education (Martin *et al.*, 2016). The programme also served as a catalyst to the development of cooking skills, and there was optimism about the potential to use these newfound competencies with families after release.

GOOP made many prisoners (and, indeed, staff) ‘feel good’ due to their sense of achievement at contributing to an enhanced environment, not just for themselves but for the wider prison community. This reflects the role of gardening in developing ‘sense of place’ (Thompson, 2012) and a growing appreciation that this is an important contributor to health (Eyles and Williams, 2008). It also echoes wider research concerning mechanisms by which therapeutic nature-based activities impact well-being (O’Brien *et al.*, 2011). Furthermore, it suggests that participation was instrumental in catalysing and/or strengthening prosocial behaviours (Maruna, 2001), key to combatting social exclusion and enabling effective resettlement. It was also evident that engagement and contact with nature played an important role in improving prisoners’ positive mental well-being, both for those working within the prison grounds and for those involved in environmental outworking. This supports the biophilia hypothesis, which contends that humans have an innate affiliation with, and gain fulfilment from their connection with, the natural world (Kellert and Wilson, 1993) and also suggests that nature connections can play an important salutogenic role, promoting human flourishing (Baybutt and Chemlal, 2016). In understanding why GOOP is perceived to have impacted so positively on prisoners’ mental health and well-being, it is valuable to examine the role played by the growing process. Reinforcing the belief that gardening can deliver therapeutic and self-developmental outcomes (Lewis, 1996), the particular ethos and focus of GOOP encouraged prisoners to nurture and care and to develop a sense of pride and ownership in their work. Reflecting the observation that success with plants can lead to success in other aspects of an individual’s life (Flagler, 1995), it was evident that these roles and sentiments, so rare within prison contexts, in turn contributed to increased self-confidence, self-esteem and self-belief – perhaps going some way towards countering the disempowerment intrinsic to prison culture (Woodall *et al.* 2014b).

Reflecting on the process of promoting health within prison contexts and observations that this has largely aligned with a biomedical perspective focused on individual behaviours (ibid.), it is salient to note that GOOP achieved its impacts largely through changing physical and social environments, shifting cultures and relationships, offering new opportunities and using horticulture to bridge and ‘join-up’ public health and criminal justice agendas (Baybutt and Chemlal, 2016). It is highly relevant that impacts extended beyond the three ‘public health’ outcome areas (physical activity, healthier eating, mental wellbeing) through the development of skills and attributes linked to work-readiness and employability – identified by prisoners as crucial to reducing reoffending and understood to be pivotal in facilitating greater social inclusion and removing barriers to successful rehabilitation (Carter, 2007).

By addressing offender management priorities, it proved easier to secure buy-in from prison staff but also ensured a strong focus on wider determinants of health, often lacking in prison health promotion (Woodall *et al.*, 2014a). This evaluation thus supports the argument that prison-based horticultural programmes offer multiple benefits – including meaningful work, skills formation, job training, development of self-confidence, and fostering of responsibility, decision-making and a work ethic (Flagler, 1995) – linked to their focus on aspirations and assets as opposed to symptoms and deficits (Lewis, 1996). Closely linked to skills development, GOOP’s strong focus on co-operation and team working proved beneficial for relationship development, reflecting evaluation findings from studies focused on wider nature-based activities (O’Brien *et al.*, 2010). By helping to build trustful and respectful relationships between prisoners and between prisoners and staff, it was understood to create a more pleasant and productive living and working ethos. This relationship building was understood to be important in preparing prisoners for life ‘on the outside’, both through ‘soft skills’ development and facilitation of actual contact with community members. Alongside this, prisoners highlighted how participation in gardening had served as a point of connection and shared experience, strengthening relationships with family members.

## CONCLUSION

The study suggests that GOOP has demonstrated real success in achieving tangible benefits for prisoners’ well-being and future opportunities. Working across key agendas relating to health, education and resettlement, GOOP has been effective in demonstrating the potential of horticulture not only to impact positively on mental health, physical activity and knowledge of food/healthier eating but also to contribute to social inclusion through the development of key transferable skills, life competencies, specialist abilities and processes of socialisation. Preparation for successful resettlement and employment beyond prison – which are key determinants of future health and life chances – requires individuals to experience work, develop their work ethic and contribute effectively to a whole team. GOOP has enabled prisoners, many of whom have not previously been employed or managed to maintain employment, to be ‘work ready’, motivated and committed.

Beyond this, there are other pressing problems in the prison setting such as self-harm and suicide, which are both increasing in the UK (Prison Reform Trust, 2015). With the provision of purposeful activity recognized as important in preserving prisoners’ well-being and potentially important for reducing self-inflicted death (Leese *et al.*, 2006), GOOP offers an important contribution. Since the research study of Phase 1 of GOOP that forms the focus for this paper was completed, prison staff have reported positive stories of how, during its subsequent implementation, GOOP is turning people’s lives around and reducing self-harm. This supports the wider literature in highlighting how horticulture can provide relevant, interesting and creative purposeful activity and simultaneously be a tool to empower, motivate and promote the mental well-being of vulnerable and excluded individuals (Barton *et al.*, 2016).

The GOOP programme was also established with a vision of strengthening relationships and joined-up working within individual establishments and across the wider offender pathway. The findings suggest that at the time of the evaluation, limited headway had been made towards this vision – with many prisons operating GOOP as a discrete gardens-based project and only limited relationships having been developed between participating prisons. This challenge of developing a truly ‘whole system’ approach is not unique to prisons but echoed by the experience of other healthy settings programmes (Newton *et al.*, 2016; Whitelaw *et al.*, 2001). However, nine years after it was established, GOOP remains active across North West England, with senior support at both regional and local prison levels and with an explicit concern to work across the whole prison and to ‘join-up’ public health and offender management agendas – testament to the holistic, systems-based settings model (Dooris, 2013).

The utilization of mechanisms such as prison delivery plans to embed GOOP into operational implementation and strategic management and monitoring has enabled a more sustained focus on the need to work differently. Key to this have been GOOP’s recent integration into the North West Strategic Programme Board for the Rehabilitative Culture of Prisons as a widely recognized and valued regional response to improving health and resettlement; and a dynamic GOOP network, bringing together prisons and other stakeholder organisations to share practice and offer peer support while also offering guidance to ensure practice meets strategic objectives of health and justice sectors.

With innovation and ‘new ways of working’ being key to the prison reform agenda (Ministry of Justice, 2016), GOOP sits well within an environment where high incidences of substance misuse, self-harm and suicide in prisons provide an increased imperative for prison governors and health-focused partner agencies to identify innovative and alternative ways to intervene. Prisoners are expected to have access to the same range of health provision as those in the wider community, including public health and health promotion. The current policy focusses on the value of green space interventions as health interventions – thereby using and engaging with the environment in a positive way and enabling the development of positive relationships with staff, prisoners and other organisations – contributes to a safe, secure and supportive prison environment.

Ultimately, good prison health is a global concern for the whole of society, with prisoners coming from and returning to the wider community (WHO Europe, 2014). Looking to the future, horticulture in prisons offers an important opportunity to connect policy agendas through enhancing learning and health literacy; building skills and enhancing employability; developing social and interpersonal skills and the competence to maintain family relationships; and promoting models of good citizenship (Baybutt and Chemlal, 2016). However, for this to have a meaningful and sustained impact on health inequalities and social exclusion within the UK and across the globe, policy – supported by further research – has a key role to play in overcoming the long-standing challenge of transitional and resettlement issues for prisoners and find ways to use programmes such as GOOP as catalysts for wider systemic and integrative change (Baybutt and Chemlal, 2016; Hansen-Ketchum and Halpenny, 2011).

## ETHICS

Ethical approval was granted by the university committee and National Offender Management Service. Key issues included secure storage of confidential data using password-protected and/or encrypted folders and informed consent to use quotations from interviews and focus groups – with prisoners’ identity being anonymized and staff members agreeing to be identified by generic role.
